# Understanding PM_2.5_ concentration and removal efficiency variation in urban forest park—Observation at human breathing height

**DOI:** 10.7717/peerj.8988

**Published:** 2020-05-07

**Authors:** Guoxin Yan, Zibo Yu, Yanan Wu, Jiakai Liu, Yu Wang, Jiexiu Zhai, Ling Cong, Zhenming Zhang

**Affiliations:** 1 School of Ecology and Nature Conservation, Beijing Forestry University, Beijing, China; 2 State Key Laboratory of Water Environment Simulation, School of Environment, Beijing Normal University, Beijing, China

**Keywords:** Forest, PM_2.5_, Near the surface, Meteorological factors, Removal efficiency

## Abstract

To increase our knowledge of PM_2.5_ concentrations near the surface in a forest park in Beijing, an observational study measured the concentration and composition of PM_2.5_ in Beijing Olympic Forest Park from 2014 to 2015. This study analyzed the meteorological factors and removal efficiency at 1.5 m above the ground (human breathing height) over the day in the forest. The results showed that the average concentrations of PM_2.5_ near the surface peaked at 07:00–09:30 and reached their lowest at 12:00–15:00. Besides, the results showed that the annual concentration of PM_2.5_ in the forest was highest during winter, followed by spring and fall, and was lowest during summer. The main chemical components of PM_2.5_ near the surface in the forest were SO_4_^2−^ and NO_3_^−^, which accounted for 68.72% of all water-soluble ions that we observed. The concentration of PM_2.5_ in the forest had a significant positive correlation with relative humidity and a significant negative correlation with temperature. The removal efficiency near the surface showed no significant variation through the day or year. In the forest, the highest removal efficiency occurred between 07:00 and 09:30 in summer, while the lowest occurred between 09:30 and 12:00 in winter.

## Introduction

Air pollution has become an increasingly serious concern in China in recent years, as economic development and urbanization, automobile exhaust, and coal industrial emissions have increased year after year (Li et al., 2016). Fine particulate matter (PM_2.5_), along with other major atmospheric particulate pollution, has become the most pressing air pollution concern in the country (Ma et al., 2013). PM_2.5_ refers to the particulate matter with atmospheric dynamic diameters less than 2.5 µm. Compared to other particles (PM_10_ and TSP, particulate matter with atmospheric dynamic diameters of 2.5 ∼10 µm and 10 ∼100 µm, respectively), the diameter of a PM_2.5_ pollutant is smaller, its surficial area is larger, and its transmission range is farther. Moreover, it contains more toxic substances and remains in the atmosphere, making it more difficult to remove (O’Connor et al., 2008). As a result, PM_2.5_ does great harm to human health and the environment (Viana et al., 2008). Finding ways to reduce fine particulate pollution has become a difficult hot-button issue for governments and residents in affected areas.

Forests represent important ecosystems, and forest canopies can capture particles in their complex branch structures and the stomata on their leaves. Therefore, forests have a strong role in adsorbing particulate matter and removing it from the atmosphere (Freer-Smith, El-Khatib & Taylor, 2004; Nowak, Crane & Stevens, 2006). Studying the concentration and composition of particulate matters in forests can provide a basis for innovation and research into technology aimed at reducing atmospheric particulate matter. The sequestration of atmospheric PM_2.5_ by forests mainly occurs in the canopy (Liu, Yu & Zhang, 2015), so researchers have focused most of their efforts on measuring and modelling the concentration and composition of PM_2.5_ far above the surface in the forest. Previous studies have shown that the diurnal variation of PM_2.5_ concentration at different heights in the forest includes two peaks and two troughs (Wang, Guo & Guangfa, 2014; Wang et al., 2005; Zhao et al., 2009). Furthermore, the relationships between meteorological factors and the concentration of PM_2.5_ have also been studied. Researchers have examined the relationship between large-scale climate and PM_2.5_ by using ArcGIS (Chen et al., 2016; Huang et al., 2015; Yuan et al., 2017), but few people have studied the effects of microclimate on PM_2.5_ concentration, especially in forests. The composition of PM_2.5_ in certain areas in the atmosphere has also been studied: one study that looked at the constituents of particulate matter in a forest focused on particles above the canopy and indicated that water-soluble inorganic ions, including SO_4_^2−^, NO_3_^−^, and NH_4_^+^, were the main ingredients of PM_2.5_. The sum of their mass concentrations accounted for more than 50% of the total water-soluble inorganic ions of PM_2.5_, and the concentrations of these ions increased with height (Li et al., 2014).

In general, previous studies of PM_2.5_ have focused on the concentration and constituents of the atmosphere at the height of the canopy, or in urban areas. Study in Beijing showed the PM_2.5_ concentration variations in forest canopy and aboveground, and PM_2.5_ concentration aboveground was shown high in morning and twilight (Yu et al., 2019). However, an average person breathes at a height of 1.5 m above the ground (Kenagy et al., 2016), but little interest has been shown in the particles near the 1.5 m above ground surface. The study on constituent changes in particles is also very important, as the constituent analysis could help to track contributors of PM_2.5_ (Khanna et al., 2018). Another reason for clarifying particle constituent is that inhaled particulate matter can have negative effects on respiratory and cardiovascular health, and can even damage DNA (Hong et al., 2002; Lelieveld & Poschl, 2017). Studying changes in the concentration and constituents at this height will provide theoretical support for future studies into the effects of PM_2.5_ on human breathing and health. Therefore, it is important to study the concentration and constituents of PM_2.5_ near the ground in forests to evaluate their removal efficiency.

In this paper, we selected the artificial forest in the southern part of Beijing Olympic Forest Park as the experiment site. The concentration and constituents of PM_2.5_ were collected at a height of 1.5 m during different seasons. The changes in the concentrations of PM_2.5_ and its constituents were analyzed and compared to nearby bare land to quantify the effects the forest had on these pollutants.

## Materials & Methods

### Materials and methods

#### Experimental site

Beijing is the economic, cultural, and political center of China and is famous for its struggles with air pollution. Beijing Olympic Forest Park is located in the North Olympic Park, Chaoyang District, Beijing. It covers an area of 680 ha and is the largest city park in Beijing. Its geographic coordinates are 40°01′03.73″N and 116°23′09.81″E. It has a rich variety of plants in a mixed forest, including 530,000 trees and shrubs of more than 180 species that respond differently to the four seasons (Li, Jie & Yi-Xia, 2006).

**Figure 1:**
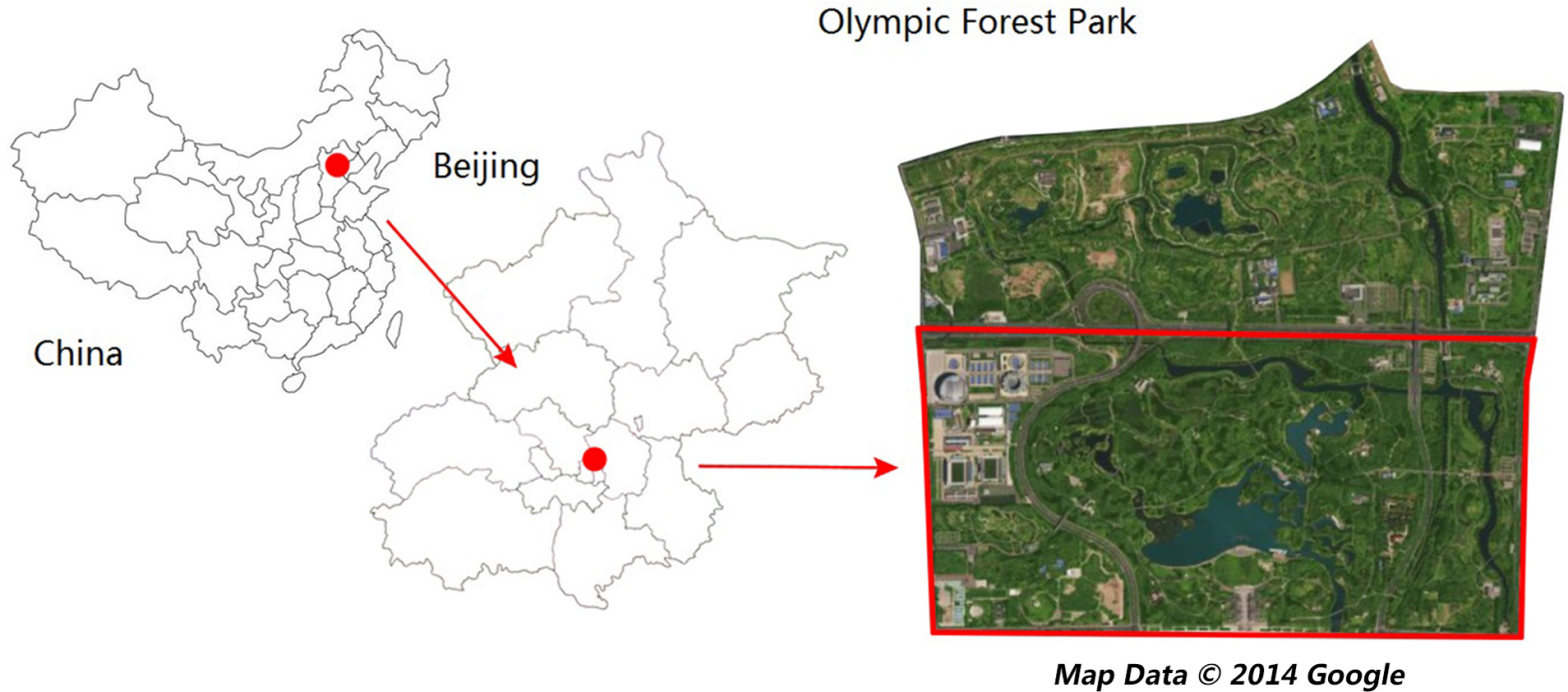
Map of the study area and sampling site locations (Olympic park image from 2014 Google Maps).

A sampling area was selected in a planted forest in the southern part of the park (Fig. 1). The sampling area was bounded by the Five-ring Road in the north and by Yang Mountain in the south. The forest was 60 m long from north-to-south and 50 m wide from east-to-west, with a stretch of pavement through the middle. The forest was mainly composed of Chinese white poplar (*Populus tomentosa*), mixed with a small amount of weeping willow (*Salix spp.*), Chinese ash (*Fraxinus chinensis*), and Chinese pine (*Pinus tabulaeformis*). Shrubs grew on the edge of the forest, including Chinese rose (*Rosa chinensis*), forsythia (*Forsythia suspensa*), and flowering peach (*Amygdalus persica*).

We selected one vertical gradient sampling location and installed equipment at 1.5 m above the ground. The composition of the underlying surface is mainly grass. There was no significant local source of air pollutants near the monitoring station. A second site was chosen as a control, which monitored the concentration over the bare land surrounding the forest. The climates were similar for the two sites. The distance between controlling point and sampling point can be around 30 m, and there were no other effects caused by people or surrounding buildings.

### Sampling procedure

**Figure 2:**
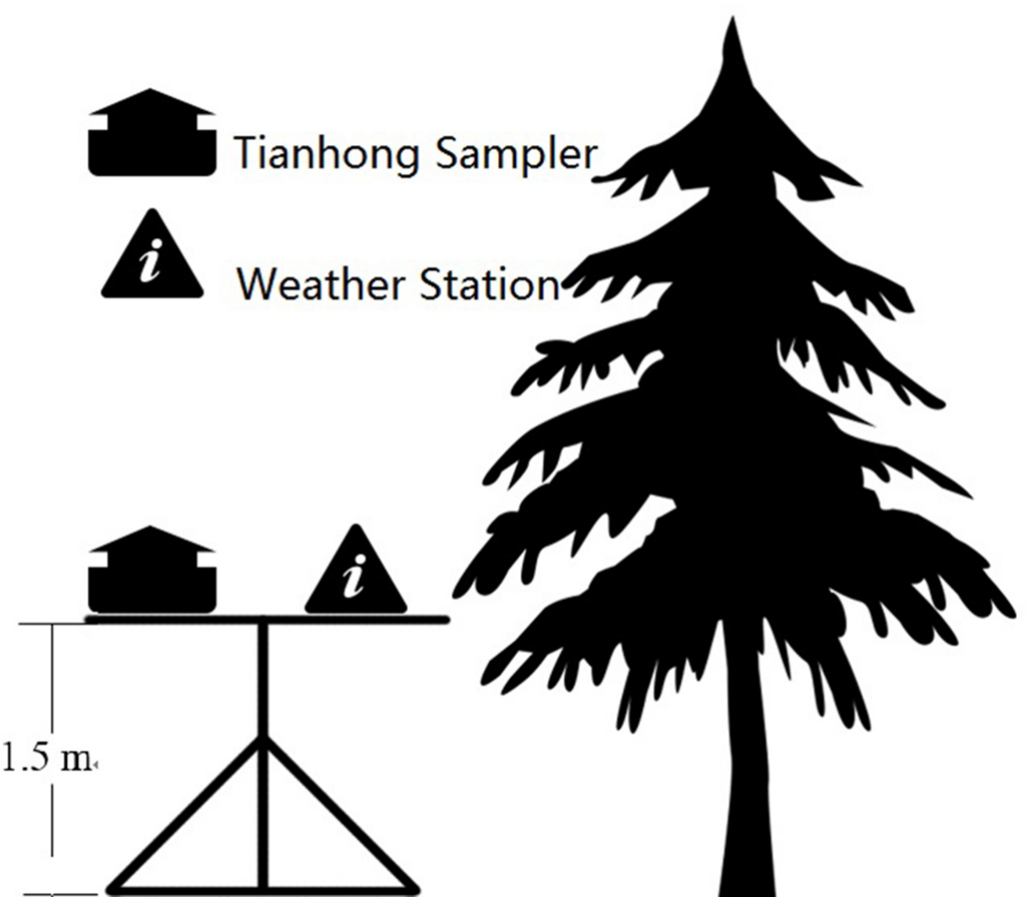
Diagram of the field installation design.

Tianhong suspended particulate pollutant sampler (TH-150C, Westernization Instrument Technology Co., Ltd, China), a handheld Dust Mate particle sampler (Turnkey Instrument Ltd., United Kingdom) and a small weather station (Kestrel 4000 Pocket Weather Meter, USA) were installed at each sampling point to collect data on particulate concentration and constituents, and meteorology (Fig. 2). Dust Mate could give us the instantaneous concentration of PM_2.5_ in every minute. The flow rate of the Tianhong sampler was set at 100 L/min, and the PM_2.5_ concentration was collected every 5 min. We used a quartz filter membrane (Whatman Grade GF/A Glass Microfiber Filter) and burned it for four h in a muffle furnace to avoid contamination and reduce error. The meteorological data collected by the weather station included temperature, relative humidity, and wind speed.

Sampling was conducted from September 2014 to September 2015. Samples were collected during the first 4 days of each month, measured from 07:00 to 18:00 at each sampling point. It provided the same sample collecting interval and we divided the sampling period into four periods which represent the morning rush hours, morning hours, afternoon hours and the evening rush hours. The weather was mainly clear during the collection period.

### Analytical methods

#### Analysis of concentration variation

The PM_2.5_ concentrations during each season were averaged by the time of day to observe the diurnal variation during each season.

#### Analysis of constituents and variation

Water-soluble ion analysis of the particulate matter was performed by soaking quarters of standard-sized portions of the sample filter membranes in 50 ml of deionized water and performing WAYEE IC6200 ion chromatography to determine the concentrations of selected anions and cations. The main constituents of PM_2.5_ were determined from these data.

#### Quantification of removal efficiency

To evaluate the importance of deposition of PM_2.5_ in a forest environment, the removal efficiency needed to be calculated. The removal efficiency differs based on several deposition-related variables, and it was calculated using the Eq. (1) (Escobedo & Nowak, 2009; Liu et al., 2016; Nowak, Crane & Stevens, 2006):

(1) \begin{eqnarray*}E=I/ \left( I+\bar {D} \right) \end{eqnarray*}

where *I* is the removed deposition of PM_2.5_ on the surface andis the daily average deposition flux. The variables I andwere calculated by Eqs. (2) and (3) with values from the forest and bare land (Escobedo & Nowak, 2009; Nowak, Crane & Stevens, 2006):

(2) \begin{eqnarray*}I=(1-R)\times {V}_{a}\times C\times t\end{eqnarray*}

(3) \begin{eqnarray*}\bar {D}=\bar {C}\times T\end{eqnarray*}

where *R* is the resuspension rate of PM_2.5_, *V*_*d*_ is the deposition velocity, *C* is the particle concentration,is the daily average concentration, and *T/t* is the evaluated time. In this process, *R* of the forest and bare land can be derived using the regression method, which can be expressed by Eq. (4) (Liu et al., 2016):

(4) \begin{eqnarray*}y=-0.01{x}^{2}+0.17x({R}^{2}=0.91,P\lt 0.001)\end{eqnarray*}

Deposition velocity depends on the surface and environment since it is related to wind speed. When the wind speed was ≥ 10 m/s, the *V*_*d*_ of PM_2.5_ was set as 2.11 cm/s; when the wind speed was <2 m/s, the *V*_*d*_ of PM_2.5_ was 0.00 to 0.09 cm/s (Nowak et al., 2013). When the wind speed was between 2 m/s and 10 m/s, the *V*_*d*_ in the forest was calculated by Eq. (5) (Nowak et al., 2013):

(5) \begin{eqnarray*}y=-0.02{x}^{2}-0.08x+0.14~({R}^{2}=0.92,P\lt 0.001)\end{eqnarray*}

The comparable equation for the *V*_*d*_ of PM_2.5_ on bare land was calculated by Eq. (6) (Liu et al., 2016):

(6) \begin{eqnarray*}y=-0.01{x}^{3}+0.05{x}^{2}+0.41x-0.05~({R}^{2}=0.98,P=0.002)\end{eqnarray*}

where *x* in the formula of Eqs. (4), (5) and (6) refer to wind speed.

## Results

### Concentration variation of PM_2.5_ in the forest

#### Diurnal variation

**Figure 3:**
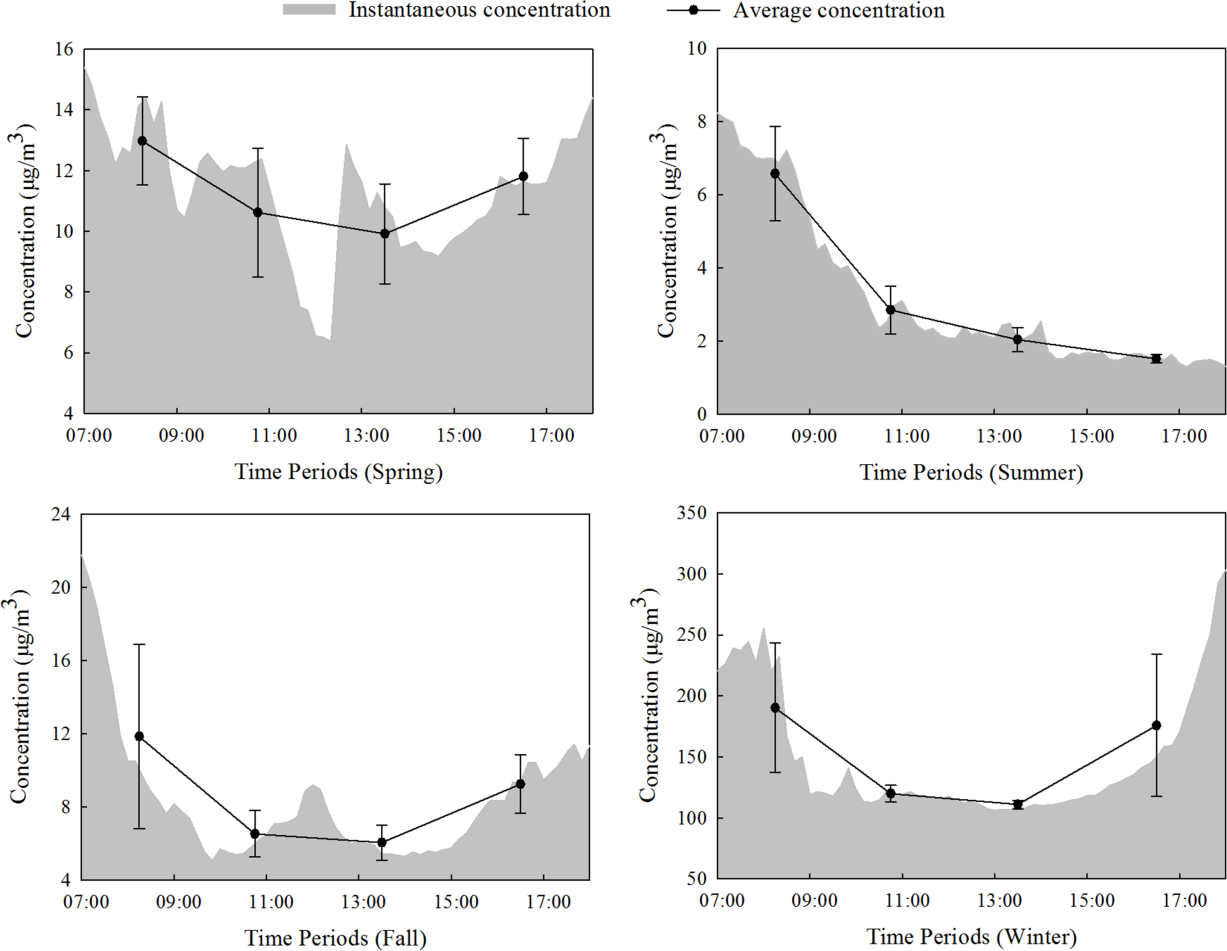
Daytime concentration changes in PM_2.5_ in the forest.

The mean mass concentration across the entire sampling period was divided into four periods: 07:00–09:30, 09:30–12:00, 12:00–15:00, and 15:00–18:00, which represent the morning rush hours, morning hours, afternoon hours and the evening rush hours. As shown in Fig. 3, the diurnal variations of PM_2.5_ concentrations in the forest were largely consistent and showed a U-shaped pattern. Summer was the exception, during which a general downward trend was observed. PM_2.5_ showed the highest average concentrations between 07:00 and 09:30. During this period, the average concentration of PM_2.5_ was approximately 12.98 µg/m^3^ in spring, 6.58 µg/m^3^ in summer, 11.85 µg/m^3^ in the fall and 190.22 µg/m^3^ in the winter. Moreover, all seasons showed the highest instantaneous concentrations at 07:00, except for winter, when the instantaneous concentration reached 303.32 µg/m^3^ at 18:00, which was the highest value during the sampling period. In the 09:30–12:00 and 12:00–15:00 periods, the average concentrations showed a relatively smooth variation, and bottomed out between 12:00 and 15:00 (except for summer), reaching 9.92 µg/m^3^ during spring, 6.06 µg/m^3^ during the fall, and 111.09 µg/m^3^ during the winter. During summer, the lowest PM_2.5_ average concentration in the forest, 1.52 µg/m^3^, occurred between 15:00 and 18:00.

#### Variations between seasons

**Figure 4:**
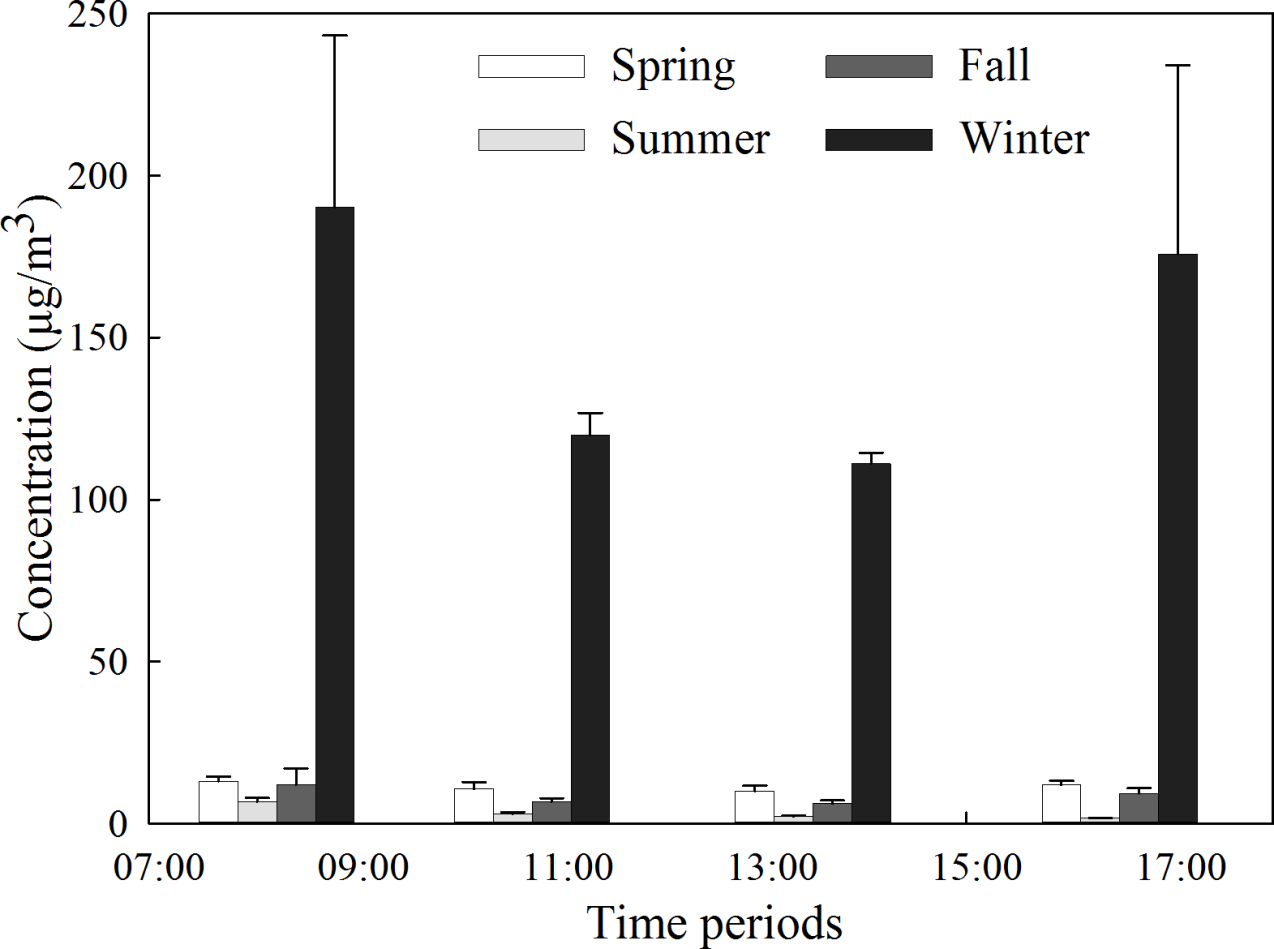
Average concentration of PM_2.5_ during different seasons in the forest.

As shown in Fig. 4, the variation in PM_2.5_ average concentrations in different periods in the forest was similar in each season. The daily average concentration of PM_2.5_ reached its highest point during winter, which was 149.31 µg/m^3^, vastly exceeding those of the other seasons. In the spring and fall, the daily average concentrations of PM_2.5_ were low, at 11.31 µg/m^3^ and 8.40 µg/m^3^, respectively. In summer, the daily average concentration of PM_2.5_ reached its minimum value of 3.17 µg/m^3^.

### Constituents of PM_2.5_ in the forest

#### Constituents and proportion of ions in PM2.5

**Figure 5:**
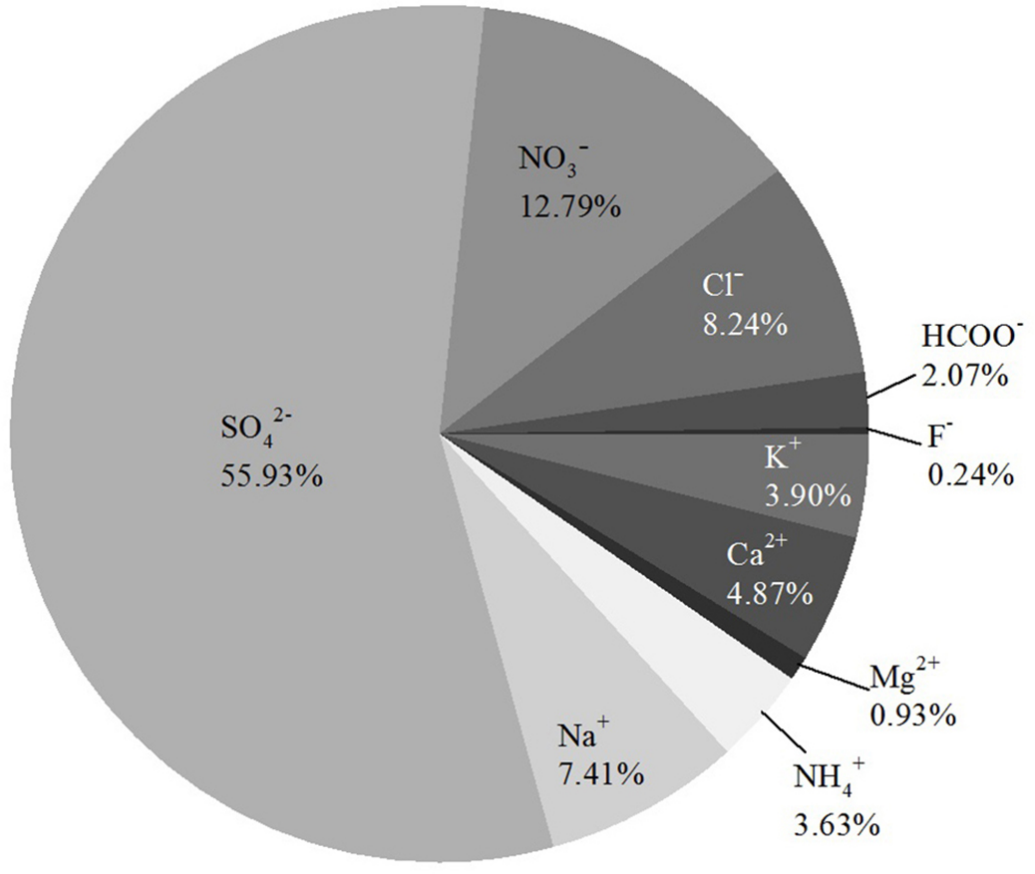
Ion composition of PM_2.5_ in the forest, by mass.

Average concentrations of ten ions were detected in this study: SO_4_^2−^(97.49 µg/m^3^), NO_3_^−^(20.50 µg/m^3^), Cl^−^(16.15 µg/m^3^), Na^+^(11.80 µg/m^3^), Ca^2+^(8.73 µg/m^3^), K^+^(6.87 *μ*g/m^3^), NH_4_^+^(7.08 µg/m^3^), HCOO^−^(3.46 µg/m^3^), Mg^2+^(1.62 µg/m^3^), and F^−^(0.44 µg/m^3^). As shown in Fig. 5, SO_4_^2−^ was most abundant, representing 55.93% of the ions, by mass, followed by NO_3_^−^, Cl^−^, Na^+^, Ca^2+^, K^+^, NH_4_^+^, HCOO^−^, and Mg^2+^. F^−^ was the least abundant at 0.24%.

#### Seasonal variations in negative and positive ions

**Figure 6:**
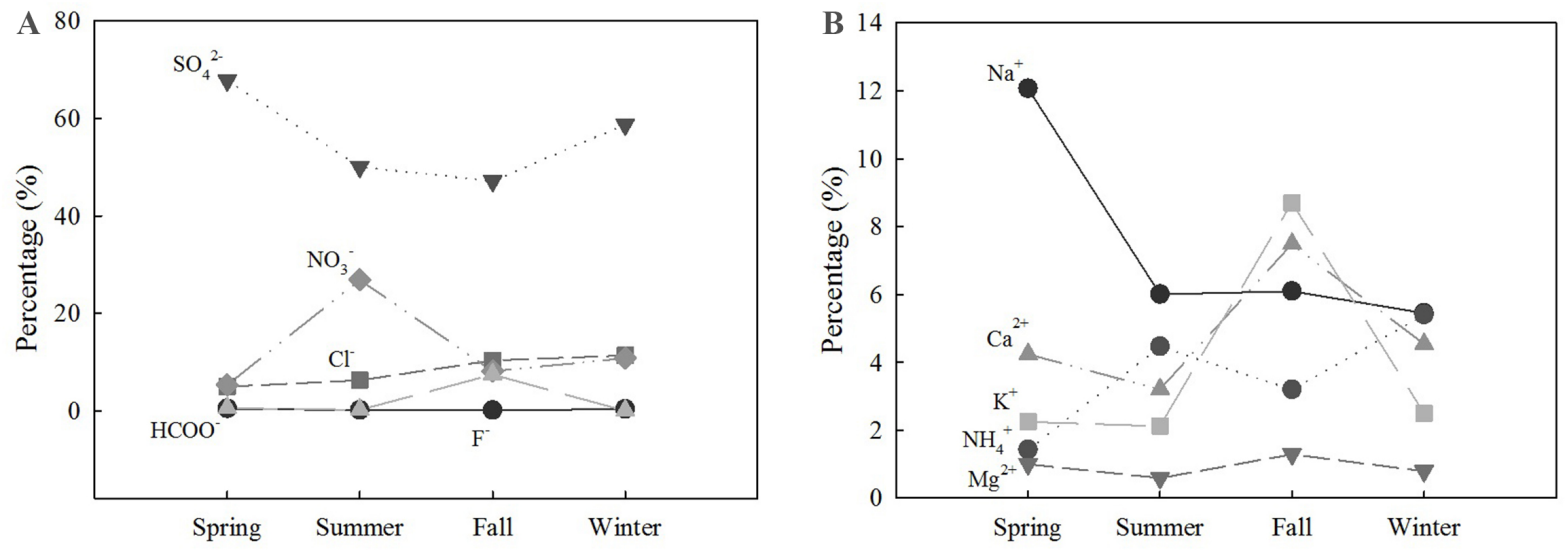
Relative abundance of anions (A) and cations (B) in the forest, across all seasons.

As shown in Fig. 6, the proportion of SO_4_^2−^ in fine particular matter showed a U-shaped variation across seasons. NO_3_^−^ experienced an obvious spike during summer. Na^+^ showed a downward overall trend and had a sharp decrease between spring and summer. Ca^2+^ and K^+^ had the same trend and both peaked during the fall. Cl^−^ and NH_4_^+^ showed an upward trend, while the remaining ions did not show significant changes in relative abundance.

### Influence of meteorological factors on PM_2.5_ in the forest

**Figure 7:**
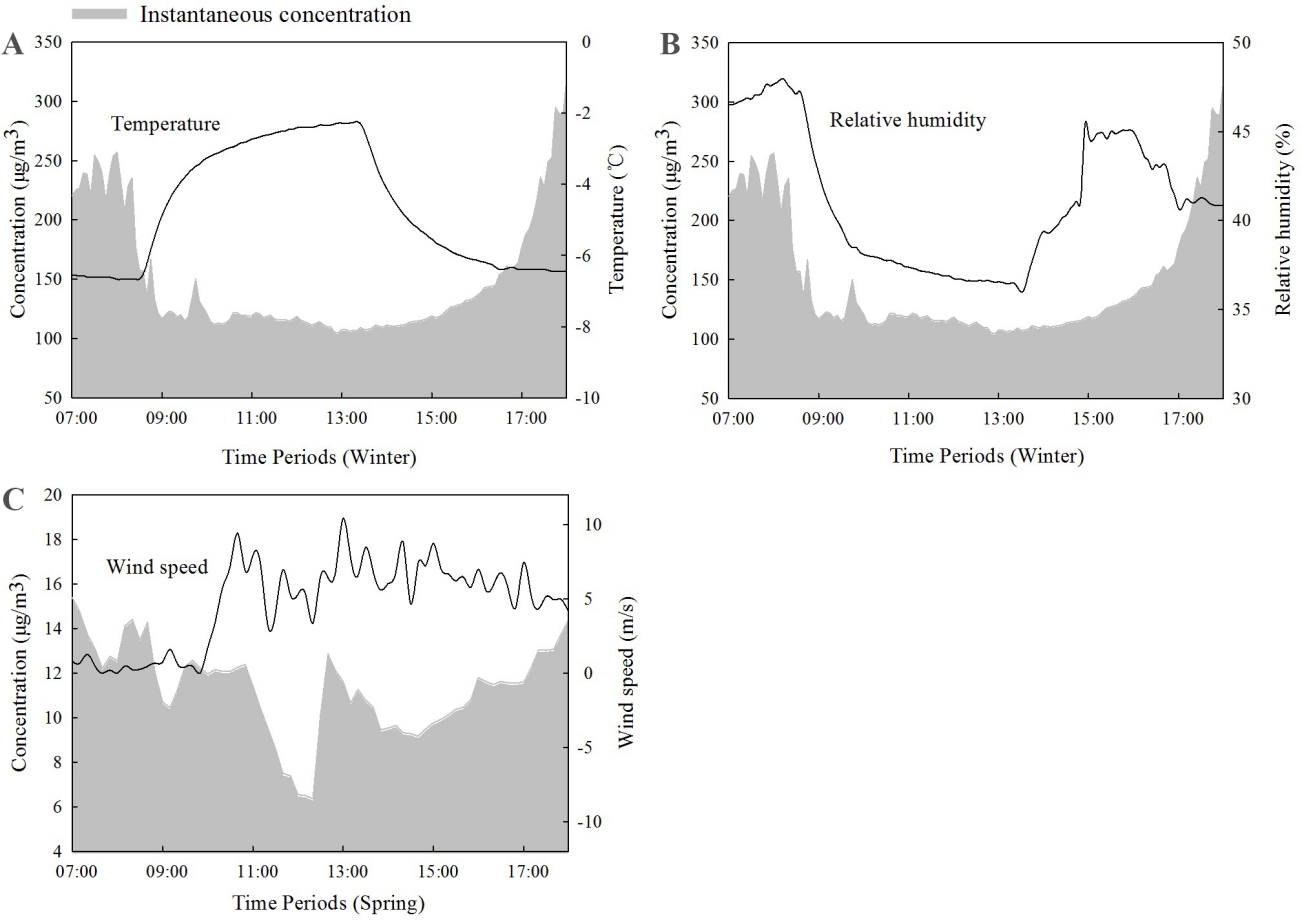
Plots showing PM_2.5_ concentrations and meteorological measurements: (A) air temperature and (B) relative humidity during the winter and (C) wind speeds during the spring.

Meteorological factors, including temperature, relative humidity, and wind speed, were recorded during each period by a small weather station. As the PM_2.5_ concentration was significantly higher during winter than during other seasons, we chose the data from winter to be representative for most meteorological variables. The exception was wind speed, which was negligible during the winter, but was relatively high during the spring. Thus, we used the data from the spring to analyze the relationship between wind speed and concentration (Fig. 7). Temperature, relative humidity, and wind speed were all correlated with the concentration of PM_2.5_ (Table 1). The concentration of PM_2.5_ had a significant positive correlation (*p* < 0.01) with relative humidity and a significant negative correlation with temperature. The correlations between the concentration of PM_2.5_ in the forest and relative humidity and temperature were particularly strong.

### Removal efficiency of PM_2.5_ in the forest

**Figure 8:**
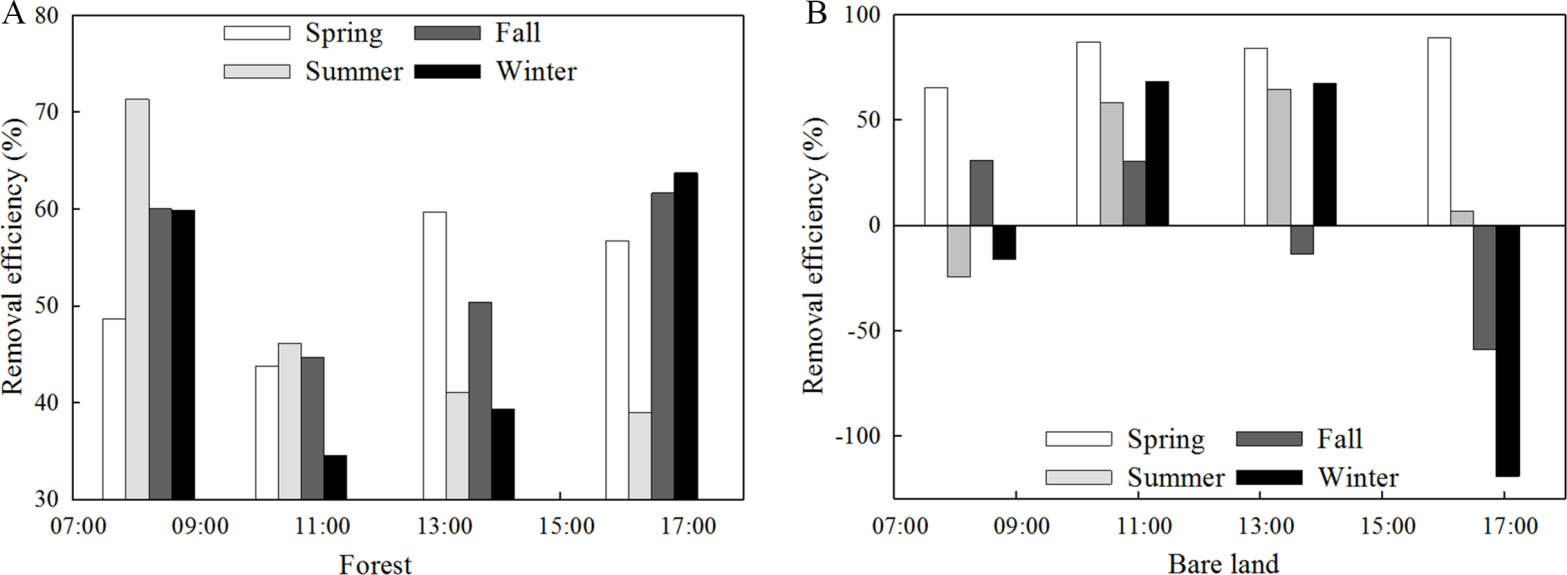
Removal efficiencies of PM_2.5_ in the forest (A) and over bare land (B) during different seasons and time periods.

The removal efficiency of PM_2.5_ during different periods was calculated according to the removal efficiency model. Figure 8 shows the PM_2.5_ removal efficiencies of the forest and bare land during each daily period. The removal efficiency of PM_2.5_ in the forest was relatively consistent throughout the day for each season. The variation in the removal efficiency for the four seasons between 07:00 and 09:30 had the same relative trend as that between 09:30 and 12:00. Summer had the highest removal efficiency between 07:00 and 09:30 and winter had the lowest between 09:30 and 11:00, which were 71.3% and 34.5%, respectively. For bare land, the removal efficiencies were significant during spring, but the other seasons varied considerably, even registering negative removal efficiencies during some periods and seasons.

## Discussion

### Concentration and removal efficiency of PM_2.5_ in different seasons and periods in the forest

Our monitoring results showed that the highest concentrations of PM_2.5_ near surface in the forest were observed in the morning. At this time, the forest still had high relative humidity and the traffic was heavy. By noon, and continuing into the afternoon, the concentrations decreased. Around dusk, the traffic was heavy again as commuters returned home, and the exhaust emissions increased, bringing particular matter concentrations up, as well. We analyzed the concentration differences of PM_2.5_ near the surface during different seasons. We showed that over a year, the concentration of PM_2.5_ near the surface in the forest was highest during winter and lowest during summer. Besides, the concentration of PM_2.5_ near the surface during winter was significantly higher than in other seasons. On the one side, that was because of winter heating in North China via the provision of coal for boilers, leading the maximum concentrations of PM_2.5_ a d PM_10_ in the winter (Zhang et al., 2015). What’s more, wintertime submicron aerosols showed an average 52% enhanced organics higher than that of summer (Sun et al., 2013). On the other side, the poor air quality measured during winter was not caused entirely by an increase in the severity of anthropogenic pollution during this season. Another important factor was the relative function of the forest. A forest canopy can capture particulate matter with its branches, leaves, and pores, thereby reducing the concentration of particulate matter in the atmosphere (Freer-Smith, El-Khatib & Taylor, 2004; Nowak, Crane & Stevens, 2006). In the winter, deciduous leaves, which are prevalent in our forested study location, had withered and fallen, and the air humidity was low. As a result, the adsorption of PM_2.5_ by the forest was low. In contrast, the canopy was thick during summer and the air humidity was high, so the removal effects of the forest were prominent. The concentration of PM_2.5_ in the forest was the lowest and, the air quality was at its best, during summer. This result reflects the capacity of forests to regulate and intercept PM_2.5_ during the year. This conclusion is consistent with many studies. For example, (Yang et al., 2002b) studied the variation in PM_2.5_ concentration and its correlation with PM_10_ and total suspended particulates in Beijing in 2002, and they found that the concentration of PM_2.5_ had clear seasonal variations, with the highest concentrations during the winter and the lowest concentrations in the summer. Many other studies (Balestrini et al., 2007; Escobedo & Nowak, 2009; Escobedo et al., 2008; Prajapati & Tripathi, 2008) have shown similar trends.

**Table 1:** Regression analysis relating meteorological factors and concentrations of PM_2.5_ in the forest.

Parameter	Meteorological factors		
	Temperature	Relative humidity	Wind speed
R^2^	0.4942	0.3326	0.1825
*p*-Value	<0.0001	<0.0001	0.0003

Table 2 shows the PM_2.5_ removal efficiencies during different seasons and periods near the surface in the forest. These removal efficiencies are based on the deposition flux (F) and the average concentration; however, deposition is influenced by the deposition velocity (V_d_), which is related to the wind speed. The uncertainty in the data possibly stems from the fact that the parameterization did not consider the processes of upward flux or rain, nor did it account for measurement uncertainties. A study has found that the blocking effects in the forest were much better under lower air quality grades (Cong et al., 2018), which means PM_2.5_ might stay in the canopy under higher PM concentration. Thus, the removal efficiency in Fig. 8 was not significantly different from other seasons in the overall trend. But what we showed in Table 2 could indicate that the removal efficiency was lower than other seasons in afternoon hours during winter. Some conclusions can still be drawn. Summers in the forest experienced the highest removal efficiency between 07:00 and 09:30 when the traffic was heavy and the concentration of PM_2.5_ was relatively high. The thick summer canopy had better adsorption rates than at other times during the day or in other seasons. Winter had the lowest removal efficiency between 09:30 and 12:00. At this time, the morning rush hour had passed but the concentration of PM_2.5_ remained high. The poor absorption rates, reduced by the lack of foliage and low humidity, contributed to the low removal efficiency. For the bare land, the removal efficiencies near the surface were influenced by the wind and the herb layer. Herb layers can help reduce dust pollution caused by wind erosion, which carries particulate matter back into the air (Liu, Yu & Zhang, 2015). Wind speed could be higher in winter in Beijing (Zhang et al., 2015). According to research about effects of wind to particles, larger wind speeds produce more violent fluctuations, which the particles tended to be resuspended. And it will have a greater effect on small particles than on large particles (Harris & Davidson, 2008), which led to negative removal efficiency values.

**Table 2:** PM_2.5_ removal efficiencies in different seasons and time periods in the forest.

Seasons	Time Periods			
	07:00–09:30	09:30–12:00	12:00–15:00	15:00–18:00
Spring	48.63%	43.73%	59.68%	56.68%
Summer	71.31%	46.12%	41.00%	38.93%
Fall	60.01%	44.64%	50.30%	61.65%
Winter	59.84%	34.53%	39.28%	63.69%

### Constituents and ions changes of PM_2.5_ in the forest

**Figure 9:**
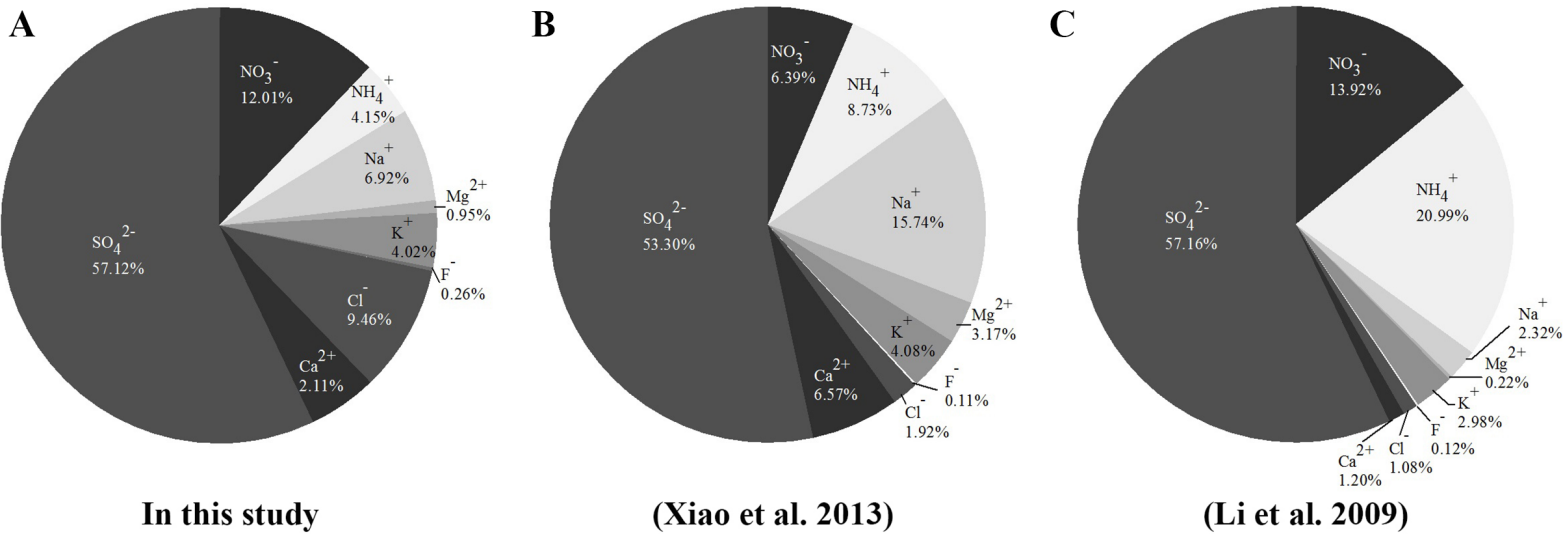
Major ion components of PM_2.5_, by mass, measured in recent years. (A) In this study; (B) Xiao et al. (2013); (C) Li et al. (2009).

The chemical composition of PM_2.5_ has been received considerable attention. The composition and sources of PM_2.5_ have been studied extensively, and SO_4_^2−^, NO_3_^−^, and NH_4_^+^ have been demonstrated as the major ions in PM_2.5_ in Beijing (Xu et al., 2007). In our study, ten ions were observed near-surface. Most water-soluble ions had similar proportions to those measured in previous studies (Fig. 9). In our study, the concentration of Na^+^ near the surface was strikingly high during the spring (Fig. 6). In coastal cities, Na^+^ in the atmosphere is mostly derived from the ocean (Xiao et al., 2013). In addition to differences between sampling sites and measurement errors, dust may also cause high concentrations of Na^+^ in PM_2.5_ (Gao et al., 2011), which may help explain our results.

The mass ratio of [NO_3_^−^]:[SO_4_^2−^] is used as an indicator of the contribution of mobile and stationary sources of nitrogen and sulfur in the atmosphere (Arimoto et al., 1996). If the ratio is greater than one, then mobile sources should be considered the main pollution sources for the sampling area, and vice versa. In our study, the ratio of [NO_3_^−^]:[SO_4_^2−^] during the sampling period was 0.21, which implies that the main pollution sources for the near-surface atmosphere in the forest were stationary. The ratio measured in this study was lower than ratios from Xiamen (0.51) (Gao et al., 1996), Changsha (0.31) (Li et al., 2007), Guangzhou (0.79) (Tan et al., 2009), Beijing (0.83–0.87) (Zhang et al., 2013), Beijing (0.67) (Wang et al., 2005), Beijing (1.00), and Nanjing (>1) (Fang et al., 2016). In those studies, sampling sites were mostly set on the roofs of buildings, while ours was near the surface in a forest, where vehicle exhaust was presumably blocked. Thus, the mass ratio of [NO_3_^−^]:[SO_4_^2−^] in our study was lower than others’. However, the influence of vehicle exhaust still can’t be ignored given the rapid increase in the number of motor vehicles in Chinese cities. Nevertheless, as we only collected on some water-soluble ion in our research, the organics in air pollution can also affect PM_2.5_ concentrations a lot. And further research should be followed.

### Influence of meteorological factors on PM_2.5_ near surface in the forest

A study about the influence of meteorological factors on PM_10_ and PM_2.5_ in Beijing found that the concentrations of particulate matter were most affected by humidity and temperature, followed by wind speed (Luo et al., 2013), which is consistent with the results of our study. However, other studies had yielded different results. Bi, Shi & Liu (2013) performed a correlation analysis of meteorological factors and PM_2.5_ in Kunming, and they found that the order of influence of meteorological factors was relative humidity >wind speed >atmospheric pressure >temperature. The following reason may account for these research finding discrepancies: the sampling sites were different, with our sampling site near the surface in a forest, where the wind speed was naturally lower. And the temperature was positively correlated with O_3_ (Zhang et al., 2015), thus, different O_3_ situation in different cities may affect the influence of meteorological factors. The PM_2.5_ concentration was also affected by various factors such as the atmospheric conditions and the source (Wu et al., 2018). Thus, the different results may be caused by the background environments of the different study sites. Our results were similar to the study of Deng in Beijing (Deng et al., 2019). Their study had a rather large time which covered four different seasons in green land and they showed the relationship between meteorological factors and PM concentration in the urban green land. It also indicated that the PM concentration was significantly positively correlated with the relative humidity (*P* < 0.01), and was significantly negatively correlated with temperature (*P* < 0.05). The PM_2.5_ concentration had the positive correlation to relative humidity during winter, and it was confirmed not only an occasional phenomenon in Beijing but also in other regions of China such as Yangtze River Delta region (Cheng et al., 2015). With the increase of relative humidity and PM_2.5_, water-soluble components became more abundant (Cheng et al., 2015).

## Conclusions

In the daytime, PM_2.5_ concentrations near the surface in the forest of Beijing Olympic Forest Park showed the highest average concentrations between 07:00 and 09:30. The lowest average concentrations occurred between 12:00 and 15:00, except during the summer, which reached its average daily low values between 15:00 and 18:00. Over a year of measurements, the concentrations were the highest in the winter and the lowest in the summer, and the concentration during the winter was significantly higher than in other seasons. The constituents of PM_2.5_ near the surface in the forest were dominated by SO_4_^2−^(55.93%) and NO_3_^−^(12.79%). The concentrations of PM _2.5_ near the surface had a significant positive correlation with relative humidity and a significant negative correlation with temperature, but no significant negative correlation with wind speed. The removal efficiency showed significant changes over the day. For the forest, the highest removal efficiency occurred between 07:00 and 09:30 in the summer while the lowest occurred between 09:30 and 12:00 in the winter. The removal efficiency of bare land was significantly lower than that of the forest; in some periods and seasons, the removal efficiency was even negative.

##  Supplemental Information

10.7717/peerj.8988/supp-1 Supplemental Information 1 Raw data of PM_2.5_ concentration, composition, meteorology, and removal efficiency

## Additional Information and Declarations

### Data Availability

The following information was supplied regarding data availability: The raw measurements are available as a Supplemental File.
